# Extracellular matrix remodeling proteins as biomarkers for clinical assessment and treatment outcomes in eosinophilic esophagitis

**DOI:** 10.1186/s12876-023-02977-z

**Published:** 2023-10-16

**Authors:** Martin Pehrsson, Willemijn E. de Rooij, Anne-Christine Bay-Jensen, Morten Asser Karsdal, Joachim Høg Mortensen, Albert Jan Bredenoord

**Affiliations:** 1 Biomarkers and Research, Nordic Bioscience A/S, Herlev, Denmark; 2 Department of Gastroenterology & Hepatology, Amsterdam University Medical Center, Amsterdam, Netherlands

**Keywords:** Eosinophilic esophagitis, Esophageal fibrosis, Non-invasive biomarkers, Collagen remodeling

## Abstract

**Background:**

Eosinophilic esophagitis (EoE) is a chronic progressive inflammatory disease of the esophagus, characterized by extracellular matrix remodeling and fibrotic stricture formation. Disease monitoring requires multiple re-endoscopies with esophageal biopsies. Hence non-invasive methods for determining tissue fibrosis and treatment efficacy are warranted.

**Aims:**

To investigate the ability of extracellular matrix proteins in serum as potential biomarkers of tissue remodeling and clinical, endoscopic, and histological disease outcomes in adult EoE patients.

**Methods:**

Protein-fingerprint assays were used to measure neo-epitope specific fragments of collagen remodeling, human-neutrophil elastase degraded calprotectin, and citrullinated or non-citrullinated vimentin in the serum of an adult EoE-cohort. Biomarker analysis, symptoms, endoscopic features and histological disease activity (eosinophils(eos) per high-power-field(hpf)) were evaluated at baseline and after six weeks of dietary intervention.

**Results:**

Patients with a baseline (Endoscopic Reference score) EREFS fibrosis subscore ≥ 2 presented with increased fibrolysis of cross-linked type III collagen (CTX-III) (*p* < 0.01), whereas low CTX-III levels were observed in patients achieving histological remission (< 15 eos/hpf) (vs. no histological remission (*p* < 0.05). Progression of endoscopic fibrosis after intervention was associated with increased levels of type-III (PRO-C3) and -VI collagen (PRO-C6) formation (all; *p* < 0.05). A baseline EREFS inflammatory subscore ≥ 2 correlated with higher neutrophilic activity (Cpa9-HNE) at week 6 (*p* < 0.05). Moreover, increased degradation of type-III (C3M) and -IV (C4M/PRO-C4) collagens were associated with remission of food impaction after intervention (all; *p* < 0.05).

**Conclusion:**

Serum extracellular matrix remodeling proteins demonstrated potential as surrogate biomarkers for assessing histological disease remission, endoscopic fibrosis, and remission of symptoms of food impaction after diet intervention in adult EoE patients.

## Introduction

Eosinophilic esophagitis (EoE) is an allergic /immune-mediated, progressive inflammatory disorder of the esophagus and often leads to fibrosis, extracellular matrix remodeling, and stricture formation [1, 2]. After its first description in the early 1990s, the worldwide EoE incidence and prevalence have emerged at rates that outpace increased disease recognition [3–8]. Overall, the development of EoE is a multifactorial interplay of genetics, environmental, and host immune system factors involved in multiple pathways [9, 10]. The association between EoE symptoms (i.e., dysphagia and food impaction) and biological disease activity (i.e., endoscopic- and histological features) is only moderate [11]. Hence, diagnosis and disease monitoring require invasive procedures such as upper endoscopy with esophageal biopsy sampling [11]. Anti-inflammatory EoE treatments include dietary elimination of culprit foods and chronic use of medication (i.e., proton pump inhibitors and swallowed topic steroids), which should be combined with endoscopic dilation in case of strictures [12]. The treatment paradigm of EoE aims to improve symptoms and reduce eosinophilic inflammation to prevent persistent histological activity progress to esophageal remodeling and fibrostenotic complications [13, 14]. The degree of fibrosis is primarily assessed endoscopically by the presence and severity of fibrotic features, such as rings and strictures [15]. Identification of remodeling and tissue fibrosis requires endoscopy with esophageal biopsies. Considering the heterogeneity of EoE, it seems possible that future treatment requires a more individual approach, with strategies depending on EoE-endotypes being more or less fibrotic [12, 16].

Esophageal fibrosis is defined as excessive extracellular matrix deposition, particularly collagen fibers, in the esophageal lamina propria. Fibroblasts are indicated as primary effector cells in fibrosis. The T-helper type 2 (T_H_2) response against food- (and aero) allergens in EoE is characterized by immune dysregulation and epithelial barrier dysfunction [10, 17]. A vigorous inflammatory state and progressive esophageal tissue damage in EoE support the secretion of pro-inflammatory and pro-fibrotic cytokines, with the subsequent fibroblast-into-myofibroblast transition. Myofibroblasts are the principal cells of collagens and lysyl oxidase (LOX) secretion, catalyzing cross-linking of the interstitial matrix collagens such as type III collagen [18]. Moreover, myofibroblasts and inflammatory cells release proteases that activate the remodeling of interstitial matrix and basement membrane collagens [19, 20]. Previous data showed that serological biomarkers targeting protein fragments of collagen cross-linking could provide novel prognostic tools for organ fibrosis and the efficacy of treatment in other fibrotic diseases (e.g., asthma, pulmonary fibrosis, and inflammatory bowel disease) [18]. Given the clinical impact of fibrotic complications, non-invasive methods using serum biomarkers to identify EoE-endotypes and disease monitoring seem to be warranted [21–23]. Therefore, in this study, we investigated the degradation and formation of the basement membrane and interstitial matrix in the serum of EoE patients as potential surrogate markers of tissue remodeling and clinical, endoscopic, and histological disease parameters.

## Methods

### Study design and patients

We analyzed biopsy samples, serum, and data collected during a prospective dietary elimination trial in adult EoE, of which details have been described previously [24]. Patients underwent upper endoscopy with biopsies at baseline and six weeks after a Four-Food Elimination Diet (FFED) (i.e., excluding gluten, milk, soy, and eggs). Symptoms, endoscopic signs, esophageal eosinophilia, and serum biomarkers were evaluated at baseline and week six. Patients were included from the outpatient clinic of the Amsterdam UMC GI Motility Center between December 2017 and January 2020. Adults (≥ 18 years) were eligible for inclusion if EoE was diagnosed according to consensus guidelines (i.e., presence of symptoms related to esophageal dysfunction and ≥ 15 eosinophils per high-power microscopic field on baseline biopsy) [7]. Patients with severe comorbidities scored as the American Society of Anesthesiologists (ASA) Physical Classification System class IV or higher, unable to stop anti-inflammatory drugs (i.e., topical or systemic steroids, leukotriene inhibitors, or monoclonal antibodies), or recent history of major Gastrointestinal surgery or GI cancer was excluded. The study included 29 age- and sex-matched commercial healthy controls (Valley Biomedical, Winchester, VA, USA).

### Ethics approval and consent to participate

The Amsterdam University Medical Center Medical Ethics Committee provided an exemption to seek formal approval for this biomarker substudy on 01-08-2019 (W19_295#19.352). All participants provided written informed consent and were given a unique study ID to ensure anonymity. All experiments were performed in accordance with relevant guidelines and regulations.

### Study procedures

#### Clinical data and sample collection

Demographics, symptoms, and endoscopic data were prospectively recorded using standardized case report forms. Symptoms were evaluated using the Straumann Dysphagia Instrument (SDI) measure [25]. The SDI measure ranges from 0 to 9 and consists of 2 items; dysphagia frequency (0 – 4) and intensity (0 – 5). Before upper endoscopy, patients underwent a venepuncture to obtain blood samples for evaluation of serum. Serum samples were collected per standardized operating procedure with subsequent storage at – 80 °C [26]. In addition, endoscopic features of EoE were classified according to the modified Endoscopic Reference Score (EREFS) grading system [15]. Endoscopic images of the esophagus were recorded to evaluate macroscopic signs and were incorporated into a slideshow (Microsoft PowerPoint 2016; Microsoft Inc., Redmond, WA, USA). All images were coded and scored randomly according to the EREFS by one blinded gastroenterologist with expertise in EoE to minimize the risk of observer bias [15]. All endoscopic features were sub-classified as inflammatory (white exudates, edema, and furrows) and fibrotic (rings and strictures) signs [27]. Of note, in case of a fibrostenotic stricture, no endoscopic dilation was performed.

In total, six biopsies were taken from the distal (2), mid (2), and proximal (2) esophagus per standardized protocol during an upper endoscopy. An × 400 magnification was used to determine the peak eosinophil count per high power field (hpf) (an area of 0.24 mm^2^).

#### Clinical subgroup definition

Patients with a score of ≥ 3 on item-2 (dysphagia intensity) of the SDI measure were defined as having symptoms of ‘food impaction’. All patients with symptoms of food impaction at baseline were further classified into two subgroups: symptoms of food impaction (yes or no) after intervention*.*

Patients with a decrease of the EREFS fibrotic subscore from baseline to week six after intervention were classified as having a *‘regressive’ phenotype,* whereas patients with an increase or no changes of EREFS fibrotic subscore (required to have EREFS fibrotic subscore at baseline > 0) were classified as a *progressive phenotype.*

Endoscopic inflammation was further stratified into clinical subgroups of patients presenting with a *‘mild inflammatory phenotype’* (EREFS inflammatory subscore 0 – 1) and *‘severe inflammatory phenotype’* (EREFS inflammatory subscore 2 – 3). Endoscopic fibrosis was also classified in the clinical subgroups of patients presenting with a *‘mild fibrotic phenotype’* (EREFS fibrotic subscore 0 – 1) and *‘severe fibrotic phenotype’* (EREFS fibrotic subscore 2 – 4)*.*

Subgroup classification of *‘Histological remission’* was defined as a peak eosinophil count of < 15 per hpf at histological assessment at week six after intervention (yes or no)*.*

#### Biomarker assays

The biomarkers included in this study are PRO-C3, PC3X, C3M, CTX-III, PRO-C4, C4M, PRO-C5, PRO-C6, C6M,VICM, VIM, and CPa9-HNE, developed and validated at Nordic Bioscience A/S, Herlev, Denmark. The blood-based biomarkers quantify neo-epitope-specific fragments reflecting collagen remodeling and immune-cell activity (Table 1) based on solid-phase enzyme-linked immunosorbent assays (ELISA). Except for PC3X and CTX-III, which are based on the sandwich ELISA platform, the remaining biomarkers are based on competitive ELISAs. Details of the biomarker-specific protocols were previously published [28–40]. Herein is provided a brief overall description of the ELISA protocols, though variations in incubation times and temperatures, specificities of the buffers, and antibodies between each biomarker exist. Firstly, a 96-well streptavidin-coated plate (Roche Diagnostics cat. no. 11940279, Hvidovre, Denmark or Greiner Bio cat. no. 655995, Kremsmünster, Austria) is coated with a biotinylated ten amino acid coater-peptide (competitive ELISA) or catcher antibody (sandwich ELISA) diluted in a buffer containing 1% bovine serum albumin (BSA) (cat.no. a-7906, ≥ 98 purity, Sigma Aldrich) for 30 min at 20 °C with 300 rounds-per-minute (RPM) rotation. Subsequently, a serial dilution of the standard peptide, controls, and patient serum sample was added to the plate, followed by 100 µL of horseradish peroxidase (HRP) labeled primary antibody diluted in a 1% BSA containing buffer (competitive ELISA) or incubation buffer (1% BSA) (sandwich ELISA), incubating one hour at 20 °C, or three hours or overnight at 4 °C depending on the assay with 300 rpm rotation. For PC3X and CTX-III, another round of incubation was required with the HRP-labeled detection antibody. Signal generation occurred by 15 min incubation at 20 °C 300 rpm rotation with 100 µL tetramethyl benzidine (TMB, Kem-EN-Tec cat. no. 438OH, Taastrup, Denmark) or BM Chemiluminescence ELISA substrate (POD) (Roche Diagnostics, cat. no. 11582950001, Hvidovre, Denmark). The reaction between the HRP-labeled antibody and TMB was stopped by adding 100 µL of 0.18 M H_2_SO_4_. The generated signal using TMB or POD was quantified using a Spectramax M5 (Molecular Devices, San Jose, CA, USA), determining the biomarker concentration (ng/mL) by extrapolating the optical density of luminescent signal to the generated 4-parametric standard curve using Softmax Pro 7 (Molecular Devices, San Jose, CA, USA.

**Table 1:** Schematic overview of the biomarkers and their neo-epitope targets

**Biomarker**	Neo-epitope specific target	Quantifies	Measurement range (LLOQ-ULOQ)
**PRO-C3**	N-terminal pro-peptide of type III collagen	Type III collagen formation	6.10 – 50.00 ng/mL
**PC3X**	Cross-linked N-terminal pro-peptide of type III collagen	Cross-linked type III collagen formation	5.80 – 72.16 ng/mL
**C3M**	MMP-9 mediated degradation of type III collagen	Type III collagen degradation	6.52 – 183.84 ng/mL
**CTX-III**	C-telopeptide of cross-linked and protease-degraded type III collagen	Degradation of cross-linked type III collagen	0.92 – 15.94 ng/mL
**PRO-C4**	Internal epitope in the 7S domain of type IV collagen	Type IV collagen formation	24.10 – 3156.00 ng/mL
**C4M**	MMP-2, -9, -12 mediated degradation of type IV collagen α1 chain	Type IV collagen degradation	8.80 – 288.00 ng/mL
**PRO-C5**	released C-terminal pro-peptide of type V collagen	Type V collagen formation	254.5 – 5600.0 ng/mL
**PRO-C6**	C-terminal of the released C5 domain of type VI collagen α3 chain (endotrophin)	Type VI collagen formation	0.80 – 135.20 ng/mL
**C6M**	MMP-2 mediated degradation of type VI collagen	Type VI degradation	2.60—235.00 ng/mL
**VICM**	Neo-epitope of MMP-2, -8 and trypsin-mediated degradation of citrullinated vimentin	Mφ degraded vimentin	0.68 – 85.30 ng/mL
**VIM**	Neo-epitope of MMP-2 and -8 degraded non-citrullinated vimentin	Non-citrullinated vimentin degradation	1.28 – 73.70 ng/mL
**CPa9-HNE**	Human neutrophil elastase degraded calprotectin	Human neutrophil elastase degradation of calprotectin	36.30 – 1970.00 ng/mL

*Abbreviations*: *LLOQ* lower limit of quantification, *MMP* matrix metalloprotease, *Mφ* macrophage, *ULOQ* upper limit of quantification

### Statistical analysis

Statistical analysis was performed in GraphPad Prism v.9.1.1 (Graph Pad Software, La Jolla, CA, USA), MedCalc v.19.3 (MedCalc Software, Ostend, Belgium), and IBM SPSS Statistics (version 25.0) (SPSS, Chicago, USA). Descriptive statistics were used to summarize all patient characteristics. Categorical variables are described as percentages, and continuous variables are expressed as median with inter-quartile range (IQR). Baseline and after-intervention values were compared using the Wilcoxon signed-rank test for non-parametric ordinal data and McNemar’s test for categorical data. The serum biomarker levels (ng/mL) for the pre-defined clinical subgroups (“Clinical subgroup definition” section) were compared at baseline and after intervention. The mean biomarker levels of CTX-III, PRO-C3, C4M, and PRO-C4 were utilized to calculate the net cross-linked fibrolysis of type III collagen and net type IV collagen turnover through division (CTX-III / PRO-C3 and C4M / PRO-C4, respectively). The biomarkers are presented as means with standard deviation (SD) or standard error of the mean (SEM). Biomarker data between EoE patients vs. healthy controls and between clinical subgroups were compared by one-way ANOVA or unpaired t-test, dependent on the number of groups. In the case of non-parametric data, Mann–Whitney U or Kruskal–Wallis test was applied. Data were corrected for multiple comparisons using Tukey for one-way ANOVA and Dunn’s for Kruskal–Wallis test. A *p*-value of < 0.05 was considered significant. Asterisks indicate: *: *p* < 0.05; **: *p* < 0.01; ***: *p* < 0.001; ****: *p* < 0.0001; NS = non-significant difference.

## Results

### Patient characteristics

In total, 29 adult EoE patients completed six weeks of diet intervention and were included in this analysis (results of the primary data of this study were previously published [24]). A male predominance was observed (*n* = 17/29, 59%), with a median age of 36.0 (IQR 29.5 – 42.5) years. A significant reduction of the median peak eosinophil count from 50.0 (IQR 40.0 – 80.0) to 25.0 (IQR 4.5 – 40.0) was observed after six weeks of intervention (*p* < 0.0001*).* Esophageal peak eosinophil counts of < 15 per hpf (histological remission) were achieved in 10 patients (*n* = 10/29, 35%) after the diet. Symptoms (SDI-score) significantly decreased from 5.0 (IQR 4.0 – 6.0) to 2.0 (IQR 0.0 – 4.0) after six weeks (*p* < 0.0001)*.* Moreover, the total EREFS score significantly decreased from 5.0 (IQR 4.0 – 5.0) to 3.0 (IQR 1.0 – 4.0) after six weeks (*p* < 0.001)*.* Additionally, a significant reduction of both the inflammatory- and fibrotic- (EREFS) subscores was observed from baseline to week six (3.0 (IQR 2.0 – 3.0) to 2.0 (IQR 1.0 – 2.0); *p* < 0.001)) and 2.0 (IQR 1.0 – 3.0) to 1.0 (IQR 1.0 – 2.0); *p* = 0.0622), respectively. More characteristics of the EoE-cohort are listed in Table 2.

**Table 2:** Schematic description of adult EoE patients at baseline and after-intervention

	**Baseline**	**After-intervention**	***P* value**
**N**	29	29	
**Patient characteristics**			
**Age (years), median (IQR)**	36.0 (29.5 – 42.5)		
**Gender (male), n (%)**	17 (59%)		
**Clinical characteristics**			
**Peak eosinophil count, median (IQR)**	50.0 (40.0 – 80.0)	25.0 (4.5 – 40.0)	< 0.0001^a,*^
**Histological remission, *yes, n (%)***		10 (35)	
**SDI, median (IQR)**	5.0 (4.0 – 6.0)	2.0 (0.0 – 4.0)	< 0.0001^a,*^
**EREFS score (total), median (IQR)**	5.0 (4.0 – 5.0)	3.0 (1.0 – 4.0)	< 0.0001^a,*^
**Fibrotic score, median (IQR)**	2.0 (1.0 – 3.0)	1.0 (1.0 – 2.0)	0.0622^a^
**Inflammatory score, median (IQR)**	3.0 (2.0 – 3.0)	2.0 (1.0 – 2.0)	< 0.001^a,*^
**Food impaction**^**I**^** (yes), n (%)**	16 (55)	9 (31)	NS

*EoE* Eosinophilic esophagitis, *EREFS* Endoscopic features are scored according to the EREFS classification and sub-classified as i) inflammatory signs including white exudates, edema and linear furrows ii) fibrotic signs including rings and strictures, *SDI* Straumann Dysphagia Instrument; Histological remission, patients with a peak eosinophil count of < 15 eosinophils (eos) per high power field (hpf) after-intervention, *IQR* Inter quartile range

^I^Patients with a score of ≥ 3 on item-2 (dysphagia intensity) of the SDI-measure were defined as having symptoms of ‘food impaction’

^a^*P* value baseline vs. after-intervention (Wilcoxon signed rank test)

^*^P-value (two-sided) of < 0.05, indicating a significant outcome

### Increased extracellular matrix remodeling in EoE patients

**Fig. 1:**
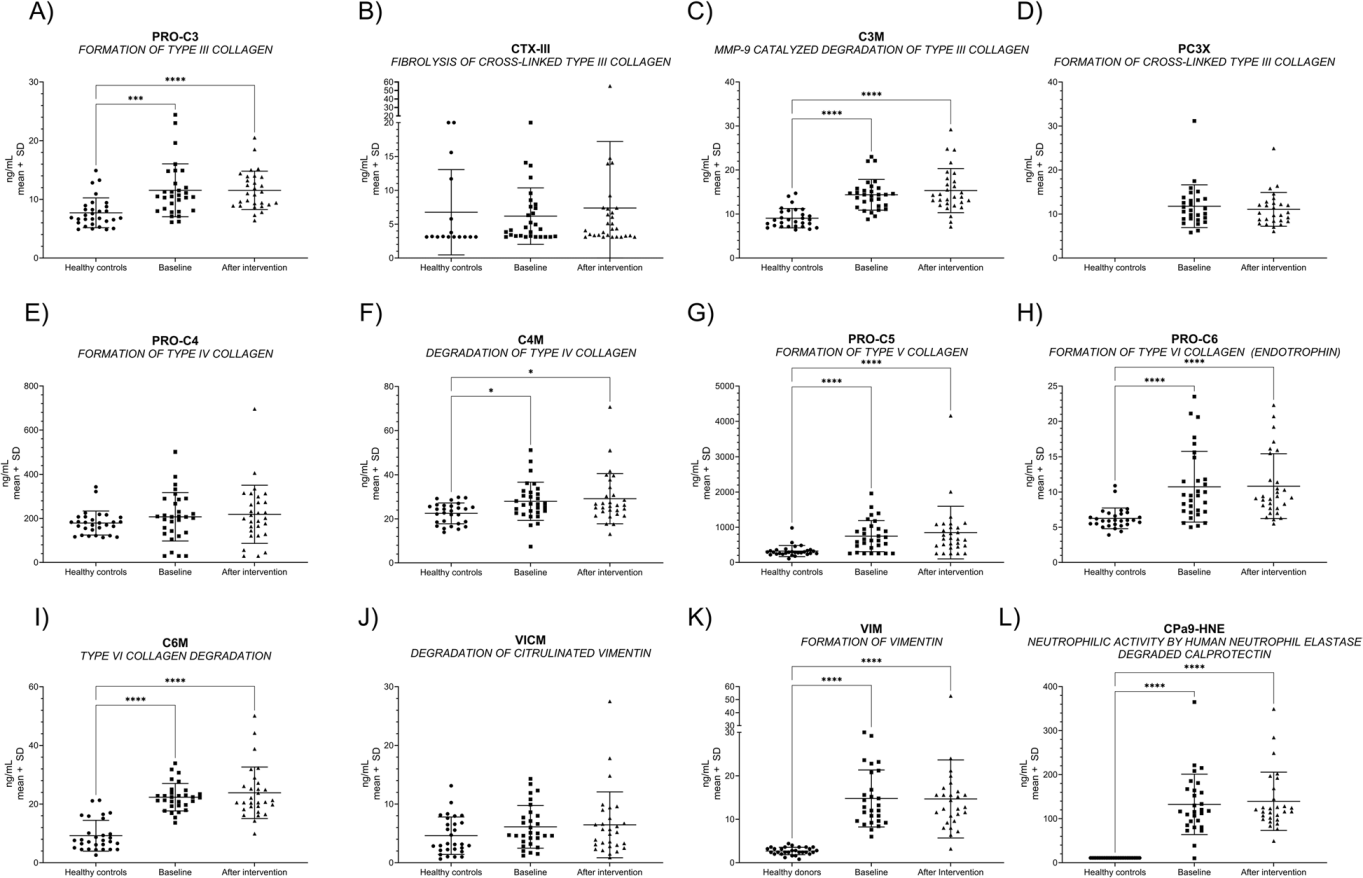
Blood-based biomarkers reflecting extracellular matrix remodeling and immune-cell activity in healthy controls and patients with EoE. The mean with standard deviation serum levels of PRO-C3 (**A**), CTX-III (**B**), C3M (**C**), PC3X (**D**), PRO-C4 (**E**), C4M (**F**), PRO-C5 (**G**), PRO-C6 (**H**), C6M (**I**), VICM (**J**), VIM (**K**), and Cpa9-HNE (**L**) were plotted for the individual patients of a group of age- and gender-matched healthy controls and patients with eosinophilic esophagitis (EoE) at baseline and after the intervention. The mean biomarker levels of patients with EoE were compared to the healthy controls using one-way ANOVA Kruskal–Wallis and applying Dunn’s test to correct for multiple comparisons. The statistical difference was calculated by unpaired t-test or Mann–Whitney, with significance as *p* < 0.05 *, *p* < 0.01**, *p* < 0.001 ***, and *p* < 0.0001 ****

Serum biomarkers (PRO-C3, C3M, C4M, PRO-C5, PRO-C6, C6M, VIM, and CPa9-HNE) in EoE patients measured at baseline and after six weeks of diet intervention showed significantly elevated levels compared to healthy controls, particularly interstitial matrix collagen remodeling biomarkers (all; *p* < 0.05). No statistical difference was observed in the overall EoE cohort’s biomarker levels when comparing baseline and after intervention levels. An overview of all measured biomarkers is presented in Table 3 and visualized in Fig. 1.

**Table 3:** Biomarker levels of healthy controls and EoE patients at baseline and after-intervention

**Biomarker**	**Controls (ng/mL)**	**EoE baseline (ng/mL)**	**EoE after-intervention (ng/mL)**	***P*-value**^a^
**PRO-C3, mean (SD)**	7.71 (2.54)	11.54 (4.51)	11.53 (3.28)	< 0.001
**C3M, mean (SD)**	9.05 (2.20)	14.38 (3.45)	15.31 (5.01)	< 0.0001
**CTX-III, mean (SD)**	6.76 (6.30)	6.19 (4.17)	7.39 (9.82)	NS
**PC3X, mean (SD)**	ND	11.78 (4.86)	11.06 (3.85)	NS
**PRO-C4, mean (SD)**	179 (54.51)	207.20 (109.90)	218.7 (132.20)	NS
**C4M, mean (SD)**	22.49 (4.73)	27.97 (8.69)	29.15 (11.39)	< 0.05
**PRO-C5, mean (SD)**	322.40 (156.30)	746.70 (440.80)	848.10 (746.90)	< 0.0001
**PRO-C6, mean (SD)**	6.26 (1.47)	10.73 (5.00)	10.83 (4.60)	< 0.0001
**C6M, mean (SD)**	9.20 (5.26)	22.34 (4.67)	23.84 (8.80)	< 0.0001
**VICM, mean (SD)**	4.60 (3.19)	6.12 (3.63)	6.46 (5.62)	NS
**VIM, mean (SD)**	2.69 (0.85)	14.79 (6.56)	14.68 (8.99)	< 0.0001
**CPa9-HNE, mean (SD)**	11.00 (0.00)^**^	132.50 (68.56)	139.40 (66.19)	< 0.0001

*Abbreviations*: *ND* not determined, *NS* non-significant

^a^Statistical differences between the measured biomarkers were calculated by two-way ANOVA applying Kruskal–Wallis correcting for multiple comparisons comparing the baseline and after-intervention biomarker levels of patients with EoE to the healthy controls. *P*-values < 0.05 were considered significant

^**^Measured CPa9-HNE levels were below the lower limit of quantification according to the assay instructions (Table 1)

### Altered interstitial matrix collagen remodeling in patients with endoscopic fibrosis

**Fig. 2:**
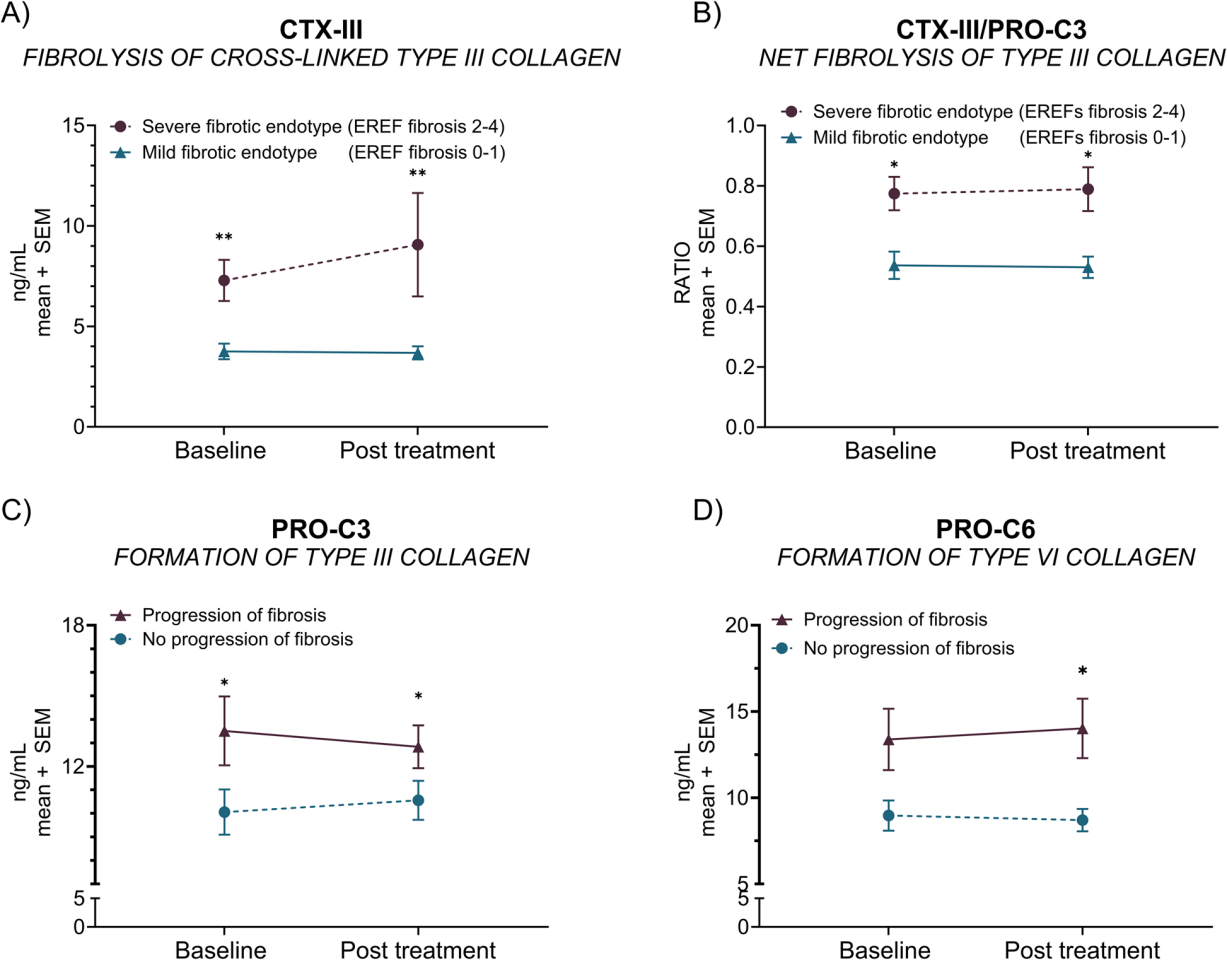
Endoscopically assessed endotypes and fibrosis changes associated with an altered interstitial matrix remodeling. Baseline and after-intervention serum CTX-III (**A**) and net cross-linked fibrolysis (CTX-III mean levels divided by PRO-C3 mean levels) (**B**) of patients with a *severe fibrotic endotype’* (EREFS fibrotic subscore 2–4; *n* = 20) or a *‘mild fibrotic endotype’* (EREFS fibrotic subscore 0–1; *n* = 9). The plotted baseline and after-intervention levels of PRO-C3 (**C**) and PRO-C6 (**D**) for patients with a *‘regressive’* (decreased EREFS fibrotic subscore; *n* = 14) or ‘*progressive’* (increased EREFS fibrotic subscore, *n* = 12) fibrotic endotype. Error bars represent standard errors of the mean (SEM). The statistical difference was calculated by unpaired t-test or Mann–Whitney, with significance as *p* < 0.05 *, *p* < 0.01**

We observed significantly higher serum CTX-III at baseline and following six weeks of diet intervention in patients with a *‘severe fibrotic endotype’* (EREFS fibrotic subscore 2–4) compared to those patients with a *‘mild fibrotic endotype’* (EREFS fibrotic subscore 0–1) (all; *p* < 0.01) (Fig. 2A). Furthermore, patients with a *‘severe fibrotic endotype’* had a high degree of cross-linked fibrolysis at week six (vs*. ‘mild fibrotic endotype’*: all; *p* < 0.05) (Fig. 2B). Patients with a fibrotic *‘progressive endotype’* (increased EREFS fibrotic subscore) had higher serum PRO-C3 at baseline and week six compared to the *fibrotic ‘regressive endotype’* (all; *p* < 0.05) (Fig. 2C). Moreover, the fibrotic *‘progressive endotype’* demonstrated significantly higher serum PRO-C6 at week six (vs. fibrotic *‘regressive endotype’*; *p* < 0.05) (Fig. 2D).

### Neutrophil activity is associated with an endoscopic ‘*severe-inflammatory phenotype*’

**Fig. 3:**
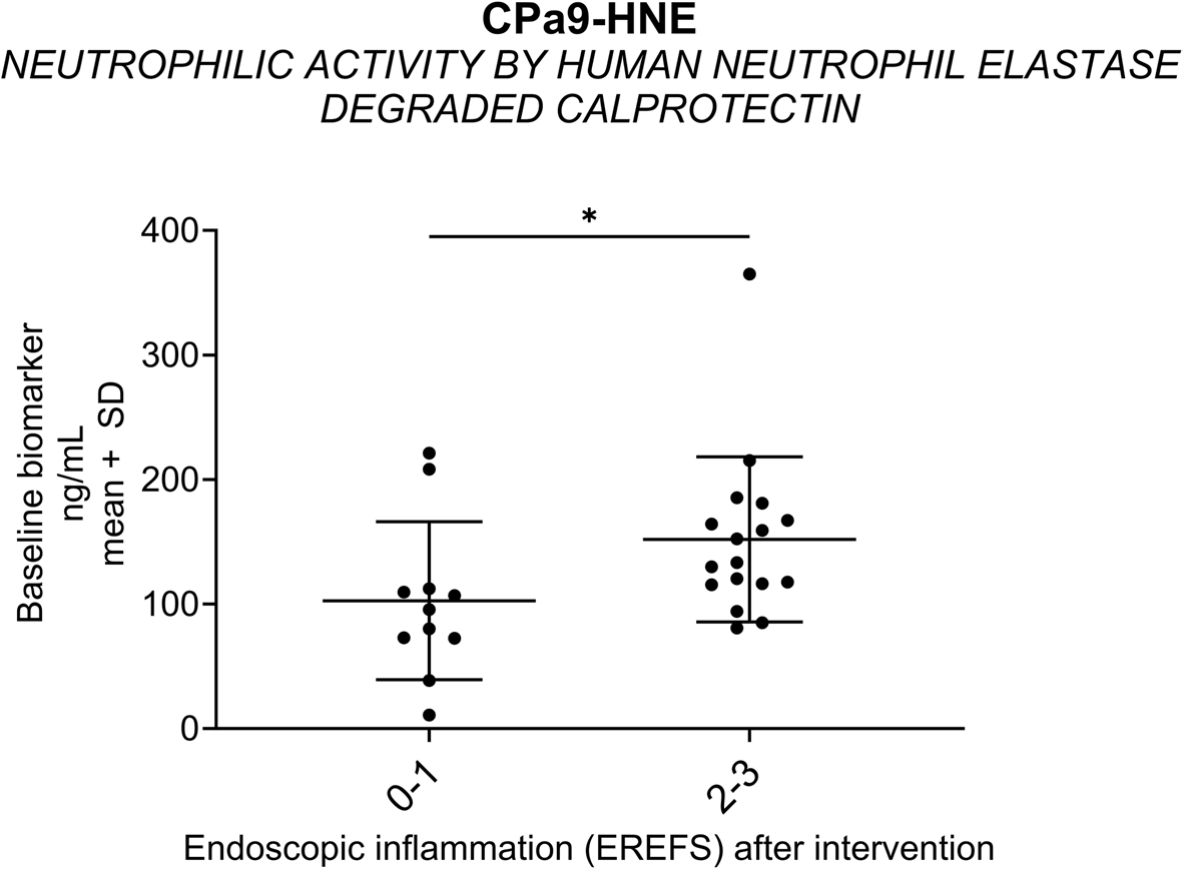
Neutrophil activity elevated with increased disease activity. Baseline biomarker levels of CPa9-HNE are plotted for patients with an EREFS inflammatory subscore of 0–1 (‘*mild inflammatory endotype’; n* = *11)* and 2–3 (‘*severe inflammatory endotype’; n* = *17)* after-intervention (**A**). The statistical difference was calculated by Mann–Whitney, with significance as *p* < 0.05 *

Patients presenting with a *‘severe inflammatory endotype’* (EREFS inflammatory subscore of 2–3) after six weeks of dietary intervention demonstrated elevated serum CPa9-HNE at baseline when compared to patients with a *‘mild inflammatory endotype’* (EREFS inflammatory subscore of 0–1) (*p* < 0.05) (Fig. 3).

### Non-invasive biomarkers of extracellular matrix remodeling are associated with histological remission and regression of food impaction

**Fig. 4:**
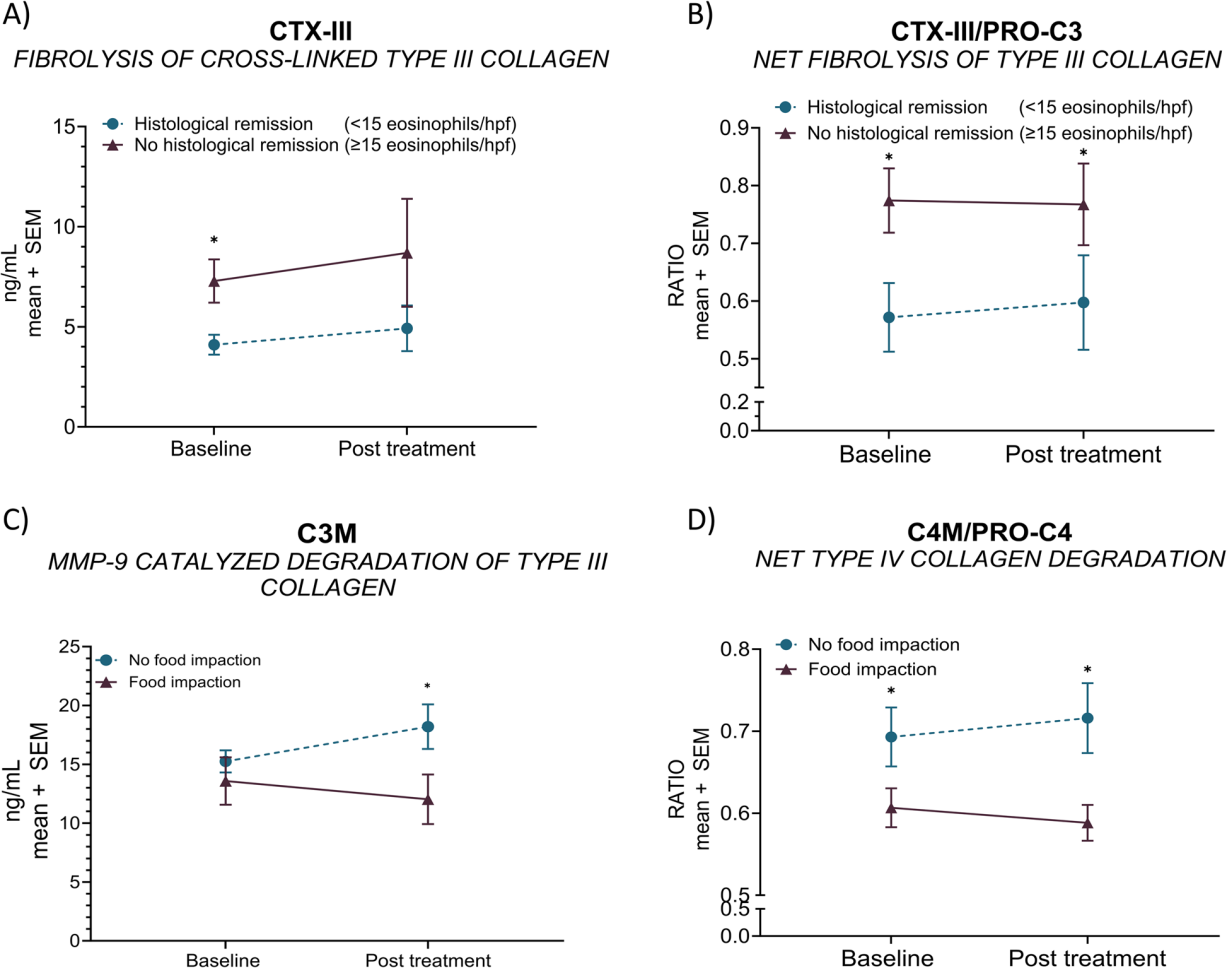
Non-invasive extracellular matrix remodeling biomarkers are associated with histological remission and regression of food impaction. CTX-III levels (**A**) and net fibrolysis (CTX-III mean levels divided by PRO-C3 mean levels) (**B**) were plotted for after-intervention ‘*histological remission’* (peak eosinophilic count < 15/hpf; *n* = 10) or ‘*no histological remission’* (peak eosinophilic count ≥ 15/hpf; *n* = 19). Biomarker levels of C3M (**C**) and net type IV collagen degradation (C4M mean levels divided by PRO-C4 mean levels) (**D**) for patients presenting with ‘*no food impaction’ (n* = *10)* or ‘*food impaction’ (n* = *6)* after intervention. Error bars represent standard errors of the mean (SEM). The statistical differences in biomarker levels were calculated by unpaired t-test or Mann–Whitney, with significance as *p* < 0.05*

Cross-linked protease degraded fragments of type III collagen (CTX-III) levels at baseline were significantly lower in patients achieving *‘histological remission’* (peak eosinophilic count < 15/hpf) vs. *‘no histological remission’* after six weeks of dietary intervention (*p* < 0.05) (Fig. 4A). Additionally, levels of net fibrolysis of cross-linked type III collagen (CTX-III/PC3X) in patients achieving *‘histological remission’* were significantly lower at baseline and after six weeks of dietary intervention compared to those patients achieving *‘no histological remission’* at week six (all; *p* < 0.05) (Fig. 4B).

Significantly higher serum levels of C3M after-intervention were observed in patients presenting with *‘no food impaction’* compared to those patients that still had symptoms of food impaction (*‘food impaction’)* at week six (*p* < 0.05) (Fig. 4C). Moreover, significantly higher levels of type IV collagen degradation at week six were seen in patients with no symptoms of food impaction (*‘no food impaction’*) after the dietary intervention (vs. symptoms of ‘*food impaction*’: *p* < 0.05) (Fig. 4D).

## Discussion

The current study presents the results of serological biomarkers directly reflecting extracellular matrix turnover (collagens) and indirectly neutrophil and macrophage activity in adult EoE patients treated with a diet. This is the first study evaluating protein fragments of degradation and formation of the basement membrane and interstitial matrix as potential surrogate serological markers of tissue remodeling and clinical, endoscopic, and histological disease outcomes in adult EoE patients.

The main key findings of our study were as follows: (1) serum biomarkers of collagen remodeling (PRO-C3, C3M, C4M, PRO-C5, PRO-C6, and C6M) and neutrophil (CPa9-HNE) and macrophage (VIM) activity are elevated in EoE patients with active disease compared to healthy controls (Table 3 and Fig. 1), (2) regression of endoscopic features of fibrosis after dietary treatment is associated with altered interstitial matrix remodeling proteins (PRO-C3 and PRO-C6) (Fig. 2) (3) Serum neutrophil activity (CPa9-HNE) is increased in patients with an endoscopic *‘severe-inflammatory endotype’* (vs. *‘mild-inflammatory endotype*’) (Fig. 3), (4) reduced serum levels of type III collagen fibrolysis (CTX-III) is observed in patients achieving *‘histological remission*’ (peak eosinophilic count < 15/hpf) after dietary intervention (vs. no histological remission) (Fig. 4), and (5) increased serum levels of type III and type IV collagen degradation were observed in those patients presenting with symptoms of food impaction prior to the diet, with no more signs of food impaction at week 6 (Fig. 4). Taken together, we uncover a potential role for serum-based extracellular matrix biomarkers reflecting the process of tissue remodeling in adult EoE.

Serological biomarkers are important to gain an objective measure of disease activity and severity and prognostic indicator and treatment outcome. Targeting neo-epitope-specific fragments originating from collagens and neutrophil activation may provide a more objective assessment of the underlying molecular processes of EoE, including tissue inflammation and fibrosis. Figure 4. provides a simplified version of the mechanism of esophageal extracellular matrix remodeling (EoE vs. healthy controls), including the suggested release of proteolytic matrix fragments to the circulation.

Compared to healthy controls, EoE patients showed an elevation of both degradation and formation biomarkers (Table 3 and Fig. 1) of the interstitial matrix (type III- and type V-collagen), type VI collagen, of the intermediate matrix between the basement membrane and interstitial matrix. Additionally, we demonstrated increased serum fragments of non-citrullinated vimentin (VIM), a protein associated with myofibroblast activity, and human neutrophil elastase catalyzed fragmentation of calprotectin. While the significant increase of these biomarkers suggests a diagnostic potential, the broad tissue expression of the analyzed proteins and their relevance in potential comorbidities minimizes their potential usage in diagnosing EoE. Though the levels of PRO-C3 in healthy controls and EoE patients were significantly different, the measured levels are within the normal range [41], severely complicating its use in diagnostics.

Additionally, biomarkers reflecting the remodeling of type IV collagen, the primary collagen of the basement membrane, did not differentiate EoE from healthy controls, in contrast to biomarkers of the interstitial matrix. The difference between interstitial matrix and basement membrane collagen remodeling suggests the interstitial matrix is the primary location of the cellular tissue remodeling process in EoE. Secretion of the interstitial matrix collagens is mainly driven by fibroblast and myofibroblast located in the lamina propria, a tissue section not always obtained during biopsies. Our data on interstitial matrix collagens indicate the importance of the blood-based biomarkers as surrogate markers of tissue remodeling in the lamina propria.

During tissue homeostasis, the collagens are constantly degraded and replaced [42, 43]. In the unresolved wound healing in EoE, there is a dysbalance between formation and degradation, where formation offsets degradation in fibrosis. Since previous data on the collagen biomarkers in other (fibrotic) diseases indicated specific neo-epitopes being associated with either fibrosis or inflammation [44–49], we sought to investigate endoscopic inflammation and fibrosis separately.

Type III collagen in the interstitial matrix is one of the most abundant collagens maintaining tissue integrity through fibril formation and LOX catalyzed cross-linking. We assessed the proteolytic degradation of cross-linked type III collagen (fibrolysis) and its formation (fibrogenesis) by measuring the CTX-III and PRO-C3 biomarkers. Our data demonstrated elevated fibrolysis in patients with a *‘severe fibrotic endotype,’* indicating an increased turnover of mature cross-linked type III collagen potentially due to excessive collagen deposition. Previous data of CTX-III in hepatitis C-associated liver fibrosis demonstrated a correlation of this biomarker with regressive liver fibrosis [39]. Patients with severe fibrosis could achieve fibrosis regression due to the resolution of the fibrotic extracellular matrix resulting from the high turnover indicated by elevated serum CTX-III. In contrast to the generation of C3M, proteolytic generation of CTX-III from mature cross-linked type III collagen could be hampered by the reduced access of MMPs to the heavily cross-linked extracellular matrix [50, 51]. However, prospective longitudinal studies are needed to evaluate this notion.

Multiple studies of organ fibrosis demonstrate the association with type III and VI collagen formation, which aligns with the data presented here [48–50]. In patients with progressive endoscopic fibrosis after intervention, we observed an increased formation of type III (PRO-C3) and type VI collagen, quantified by the pro-fibrotic endotrophin fragment (PRO-C6) [47], suggesting that these markers accurately reflect fibrogenesis. Thus, EoE patients experiencing fibrosis regression after the diet shift the balance between interstitial matrix and intermediate matrix collagens to a decreased formation of type -III and -VI collagen.

When we investigated the relationship between the biomarkers and endoscopic inflammation, the Cpa9-HNE biomarker demonstrated an association. The Cpa9-HNE biomarker assesses neutrophilic activity by quantifying neutrophil elastase-degraded calprotectin. The increased serum Cpa9-HNE levels observed at baseline associated with a *‘severe inflammatory endotype’* after the intervention suggested a potential prognostic value. The levels of CPa9-HNE are significantly lower in the EoE patients compared to previous studies of this biomarker [38]. *Bartig *et al*.* [52] demonstrated a significant correlation between platelets, eosinophils, and neutrophils in EoE. Neutrophils and epithelial cells represent potential origin cells of serum CPa9-HNE through their secretion of neutrophil elastase and calprotectin [53]. Although neutrophils are rare in EoE, the increased Cpa9-HNE levels suggest an increased activity and functional role of the neutrophils.

Furthermore, the biomarkers CTX-III and the ratio of CTX-III to PRO-C3 could differentiate between patients achieving histological remission (< 15 eos/hpf) and those who still had an active histological disease (≥ 15 eos/hpf) after the diet. The biomarker ratio, assessing net fibrolysis, enabled the optimal patient separation for histological remission. Patients who did not achieve histological remission after the diet demonstrated the highest degree of fibrolysis at both time points, suggesting the pathological association of fibrolysis in EoE. Moreover, the data indicate a potential direct or indirect association between proteolysis of cross-linked type III collagen and eosinophils. Collagens are known targets of the matrix metalloproteases (MMPs), of which eosinophils secrete MMP-2 [19], MMP-9 [54], MMP-14 [19], and potentially MMP-12 [55]. Our data on fibrolysis indicates a more severe disease course in patients with higher levels of type III collagen fibrolysis, as these patients demonstrate higher baseline fibrosis or fail to achieve histological remission after intervention. As such, these patients may be less eligible for a diet intervention.

Additionally, elevated levels of MMP-9 mediated degradation of type III collagen and net type IV collagen degradation measured at baseline and week six were observed in patients experiencing no more symptoms of food impaction after the intervention. MMP-9 is responsible for the activation of IL-1β and TGF-β [54–56], exerting both protective and pathological effects in EoE. The increased degradation of interstitial matrix type III collagen and basement membrane type IV collagen could result in the clearance of pathological extracellular matrix, alleviating fibrosis and, consequently, food impaction. Determining the degradation of the interstitial matrix and basement membrane collagens may aid in identifying patients more likely to have clinical disease remission.

Our biomarker data collectively suggest their potential as surrogate markers for monitoring treatment efficacy negating the requirement for multiple re-endoscopies with biopsies after the initial EoE diagnosis. A substantial economic burden of EoE is related to medical resource utilization costs (e.g., upper endoscopy with biopsies) [57, 58]. Thus, less invasive disease monitoring lowers healthcare costs (i.e., reduced procedures and complication risk) and improves patients’ health-related quality of life [58]. As the presented biomarkers provide an objective measure of extracellular matrix remodeling (i.e., reflecting the process of fibrolysis, fibrogenesis, and inflammation), their implementation may be important within the context of EoE-endotype identification with subsequent improvement of clinical treatment outcomes. A critical question in the development of therapeutics remains whether anti-fibrotic agents capable of modifying the natural course of EoE are warranted. Our observations suggest that stratification of EoE patients based on the biomarkers and the cellular processes associated with their release will likely assist with a more efficacious personalized (targeted) therapy selection in future practice.

Our study design has a few limitations that merit attention. First, including a small cohort of EoE patients from a tertiary healthcare center is known for limiting its statistical power and generalizability of outcomes. However, it should be noted that our EoE cohort reflects a diversified population, including different stages of disease severity. Compared to previous studies, the overall histological response rate (< 15 eos/hpf) of 35% after 6 weeks of diet seems remarkably lower. In a study by Molina Infante et al., complete histological remission was reported in 54% of EoE patients after 6 weeks of FFED [59]. It could be argued that the use of a broader elimination approach in this study, including gluten, milk, egg, and all kinds of legumes (e.g., lentil, peanut, soy) instead of only soy, which may be an explanation for these observed differences in remission rates. Moreover, the outcomes of a recent multicentre trial were also reported lower than expected remission rates in both children and adults, suggesting a potential bias in previous cohort studies as an explanation for the observed variation in results [60, 61]. Thirdly, the assessment of fibrosis was based on endoscopic features and clinical complications since a direct measure of tissue fibrosis (e.g., Trichrome staining of the lamina propria) was not available. Finally, only the effect of dietary treatment was evaluated, and the effects of other treatments may differ. However, even with this relatively small group and indirect measures of fibrosis, this is the first study until now evaluating protein fragments of collagen remodeling and neutrophil activity in serum that could serve as surrogate markers for monitoring treatment efficacy in clinical trials and practice.

In summary, this study emphasizes the clinical potential of serological biomarkers of extracellular matrix remodeling in adult EoE patients, with several of these biomarkers showing elevated levels compared to healthy controls. Biomarkers directly reflecting basement membrane and interstitial matrix turnover demonstrated prognostic potential for assessing histological disease remission, endoscopic fibrosis, and remission of symptoms of food impaction after diet intervention. Additionally, we demonstrated a relationship between neutrophil activity and the degree of endoscopic inflammation. These results provide initial insights into potential prognostic biomarkers of extracellular matrix remodeling in EoE and provide a mechanistic foundation for future studies.

## Data Availability

The datasets used and/or analysed during the current study available from the corresponding author on reasonable request.

## Abbreviations

EoE: Eosinophilic esophagitis
eos: Eosinophils
hpf: High power field
PPI: Proton pump inhibitor
Th2: T-helper type 2
LOX: Lysyl oxidase
ASA: American Society of Anesthesiologists
GI: Gastrointestinal
IQR: Interquartile range
EREFS: Endoscopic Reference score
FFED: Four-food elimination diet
SD: Standard deviation
SEM: Standard error of the mean
SDI: Straumann Dysphagia Instrument
