# Correction: Extracellular matrix remodeling proteins as biomarkers for clinical assessment and treatment outcomes in eosinophilic esophagitis

**DOI:** 10.1186/s12876-023-03048-z

**Published:** 2023-11-22

**Authors:** Martin Pehrsson, Willemijn E. de Rooij, Anne-Christine Bay-Jensen, Morten Asser Karsdal, Joachim Høg Mortensen, Albert Jan Bredenoord

**Affiliations:** 1 Biomarkers and Research, Nordic Bioscience A/S, Herlev, Denmark; 2 Department of Gastroenterology & Hepatology, Amsterdam University Medical Center, Amsterdam, Netherlands

**Correction: BMC Gastroenterol 23, 357 (2023)**

**https://doi.org/10.1186/s12876-023-02977-z**

Following publication of the original article [1] it was reported that Figs. 1, 2, 3 and 4 were either missing asterisks indicating statistical significance, or the asterisks were incorrectly replaced with a ‘1’.

The affected figure panels were the following:

• Fig. 1A, C, F, G, H, I, K, and L
• Fig. 2A-D
• Fig. 3
• Fig. 4D

**Fig. 1:**
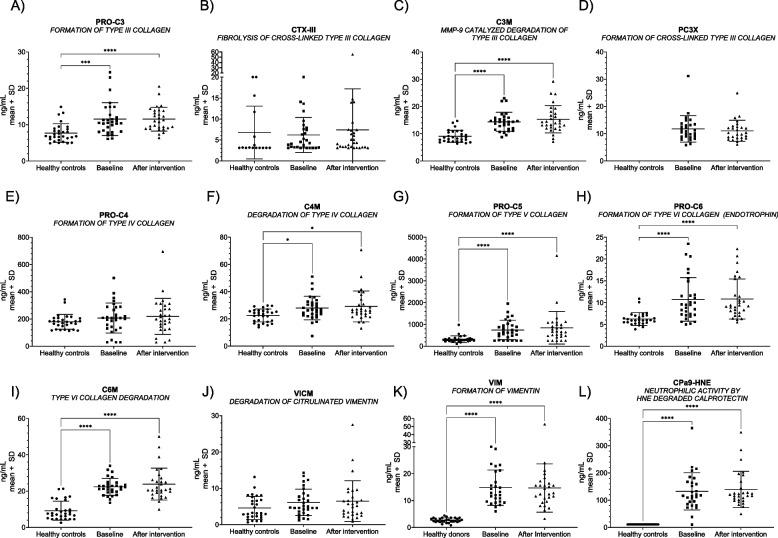
Blood-based biomarkers reflecting extracellular matrix remodeling and immune-cell activity in healthy controls and patients with EoE. The mean with standard deviation serum levels of PRO-C3 (**A**), CTX-III (**B**), C3M (**C**), PC3X (**D**), PRO-C4 (**E**), C4M (**F**), PRO-C5 (**G**), PRO-C6 (**H**), C6M (**I**), VICM (**J**), VIM (**K**), and Cpa9-HNE (**L**) were plotted for the individual patients of a group of age- and gender-matched healthy controls and patients with eosinophilic esophagitis (EoE) at baseline and after the intervention. The mean biomarker levels of patients with EoE were compared to the healthy controls using one-way ANOVA Kruskal–Wallis and applying Dunn’s test to correct for multiple comparisons. The statistical difference was calculated by unpaired t-test or Mann–Whitney, with significance as *p* < 0.05*, *p* < 0.01**, *p* < 0.001 ***, and *p* < 0.0001 ****

**Fig. 2:**
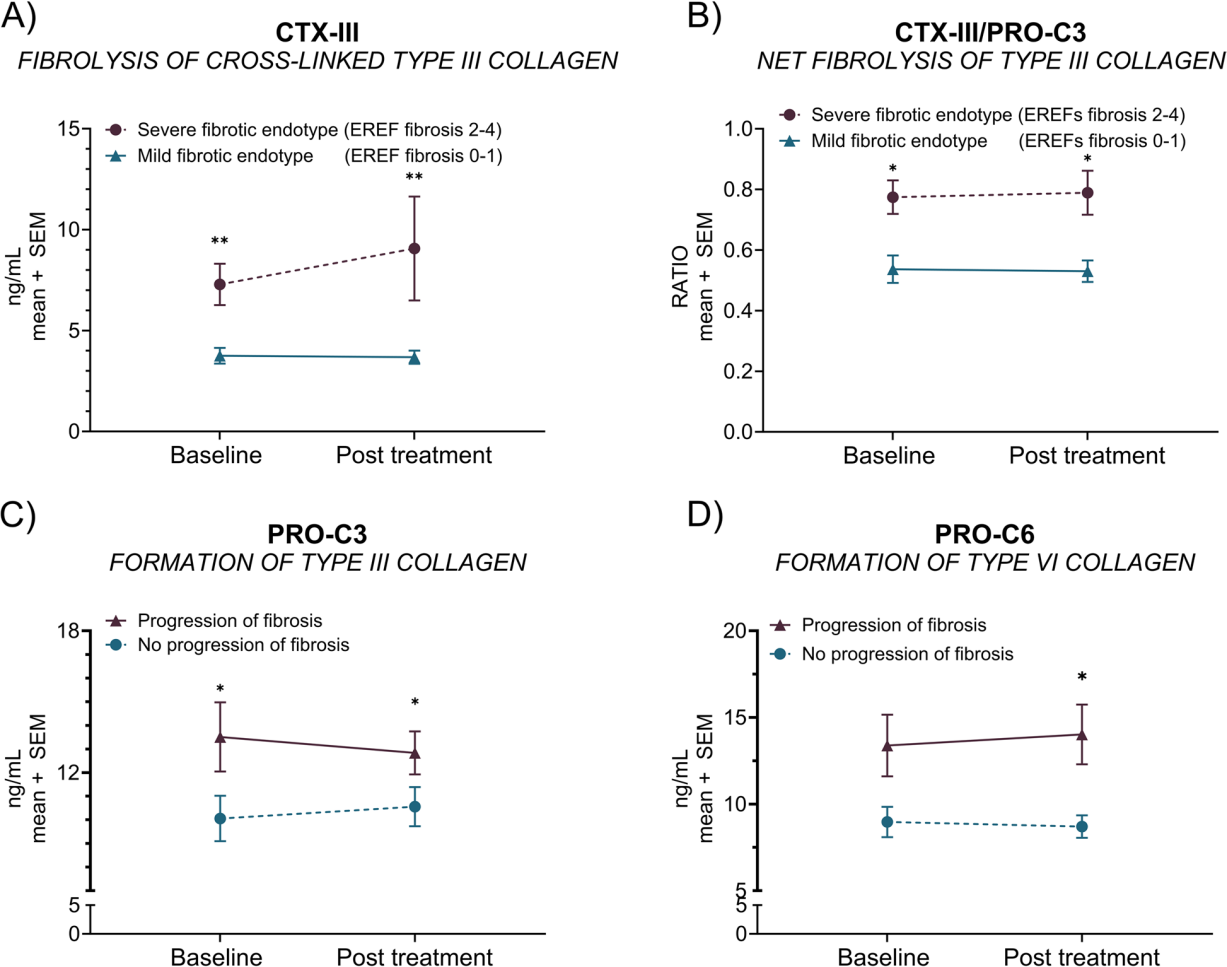
Endoscopically assessed endotypes and fibrosis changes associated with an altered interstitial matrix remodeling. Baseline and after-intervention serum CTX-III (**A**) and net cross-linked fibrolysis (CTX-III mean levels divided by PRO-C3 mean levels) (**B**) of patients with a *severe fibrotic endotype’* (EREFS fibrotic subscore 2–4; *n* = 20) or a *‘mild fibrotic endotype’* (EREFS fibrotic subscore 0–1; *n* = 9). The plotted baseline and after-intervention levels of PRO-C3 (**C**) and PRO-C6 (**D**) for patients with a *‘regressive’* (decreased EREFS fibrotic subscore; *n* = 14) or ‘*progressive’* (increased EREFS fibrotic subscore, *n* = 12) fibrotic endotype. Error bars represent standard errors of the mean (SEM). The statistical difference was calculated by unpaired t-test or Mann–Whitney, with significance as *p* < 0.05 *, *p* < 0.01**

**Fig. 3:**
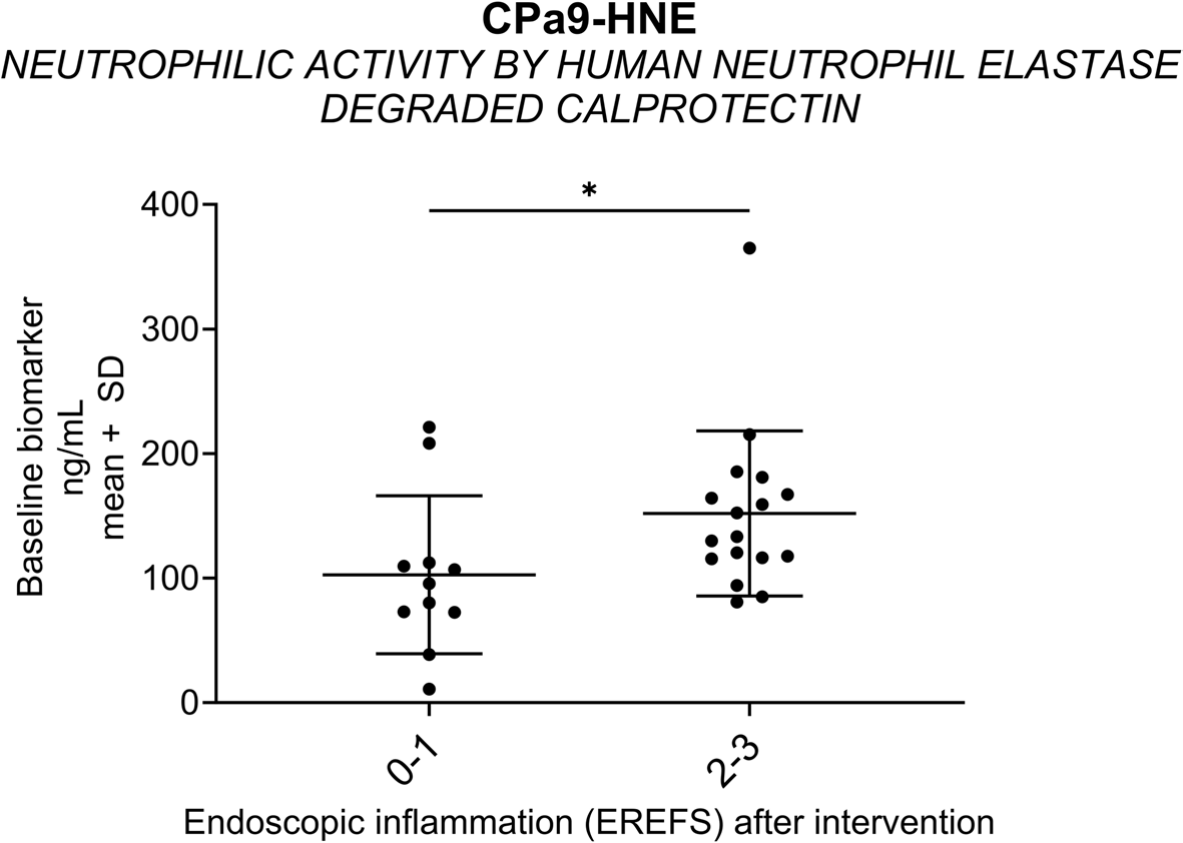
Neutrophil activity elevated with increased disease activity. Baseline biomarker levels of CPa9-HNE are plotted for patients with an EREFS inflammatory subscore of 0–1 (‘*mild inflammatory endotype’; n* = *11)* and 2–3 (‘*severe inflammatory endotype’; n* = *17)* after-intervention (**A**). The statistical difference was calculated by Mann–Whitney, with significance as *p* < 0.05 *

**Fig. 4:**
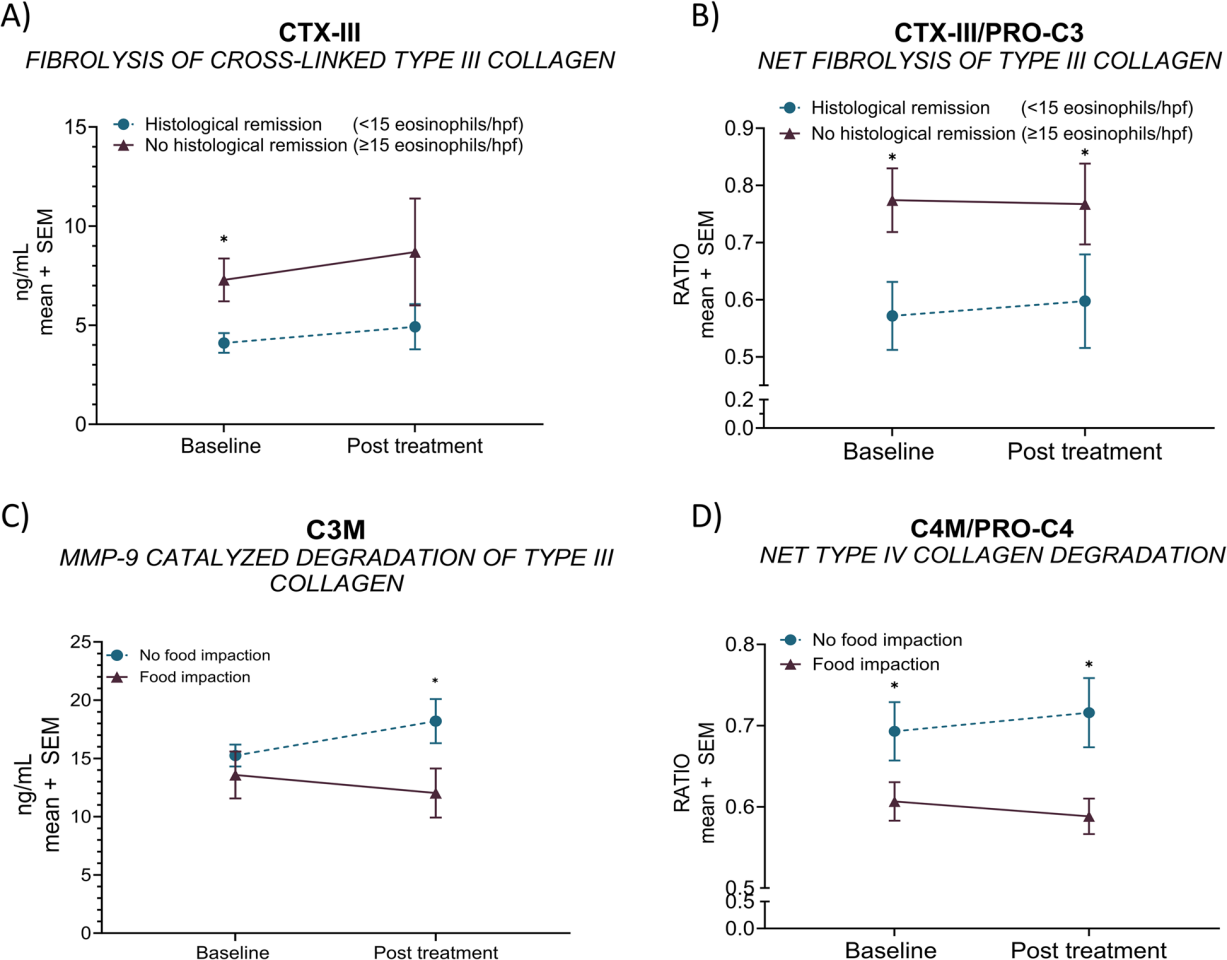
Non-invasive extracellular matrix remodeling biomarkers are associated with histological remission and regression of food impaction. CTX-III levels (**A**) and net fibrolysis (CTX-III mean levels divided by PRO-C3 mean levels) (**B**) were plotted for after-intervention ‘*histological remission’* (peak eosinophilic count < 15/hpf; *n* = 10) or ‘*no histological remission’* (peak eosinophilic count ≥ 15/hpf; *n* = 19). Biomarker levels of C3M (**C**) and net type IV collagen degradation (C4M mean levels divided by PRO-C4 mean levels) (**D**) for patients presenting with ‘*no food impaction’ (n* = *10)* or ‘*food impaction’ (n* = *6)* after intervention. Error bars represent standard errors of the mean (SEM). The statistical differences in biomarker levels were calculated by unpaired t-test or Mann–Whitney, with significance as *p* < 0.05*

The updated figures are included in this Correction article and the original article has been updated.
